# CSF Biomarkers for Alzheimer's Disease Diagnosis

**DOI:** 10.4061/2010/606802

**Published:** 2010-06-23

**Authors:** A. Anoop, Pradeep K. Singh, Reeba S. Jacob, Samir K. Maji

**Affiliations:** Department of Biosciences and Bioengineering, IIT Bombay, Powai, Mumbai 400076, India

## Abstract

Alzheimer's disease (AD) is the most common form of dementia that affects several million people worldwide. The major neuropathological hallmarks of AD are the presence of extracellular amyloid plaques that are composed of A*β*40 and A*β*42 and intracellular neurofibrillary tangles (NFT), which is composed of hyperphosphorylated protein Tau. While the amyloid plaques and NFT could define the disease progression involving neuronal loss and dysfunction, significant cognitive decline occurs before their appearance. Although significant advances in neuroimaging techniques provide the structure and physiology of brain of AD cases, the biomarker studies based on cerebrospinal fluid (CSF) and plasma represent the most direct and convenient means to study the disease progression. Biomarkers are useful in detecting the preclinical as well as symptomatic stages of AD. In this paper, we discuss the recent advancements of various biomarkers with particular emphasis on CSF biomarkers for monitoring the early development of AD before significant cognitive dysfunction.

## 1. Introduction

**Figure 1:**
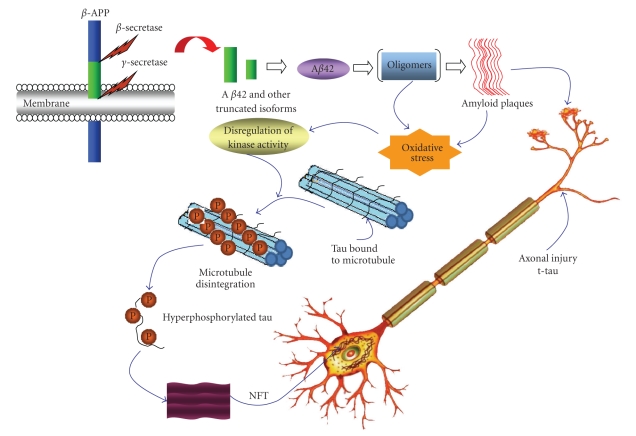
Pathological cascades and potential biomarkers of AD. Proteolytic cleavage of APP first by *β*-secretase followed by *γ*-secretase can produce A*β*42 and other shorter A*β* fragments. The subsequent aggregation of A*β*42 results in oligomers and amyloid fibrils. Amyloid fibrils are eventually deposited as senile plaques as shown. The toxicity of oligomers and amyloid fibrils could lead to the cascade of tau-hyperphosphorylation, which is otherwise bound to microtubules, providing microtubule stability. Upon hyperphosphorylation, tau dissociates from microtubules and aggregates into NFT, which could eventually cause increased cytoskeleton flexibility and neuronal death.

Alzheimer's disease (AD) is the most widespread neurodegenerative disease globally [1] and is estimated to afflict more than 27 million people worldwide [2]. AD accounts for at least 60% of all dementia diagnosed clinically. The major pathological hallmarks of AD are the loss of neurons, occurrence of extracellular senile plaques as well as intracellular neurofibrillary tangles (NFT) [3]. Senile plaques are primarily composed of amyloid *β*-protein (A*β*), which is produced from the amyloid precursor protein (APP) by sequential proteolytic cleavages made by two proteolytic enzymes, *β*-secretase (*β*-site APP-cleaving enzyme; BACE) and *γ*-secretase (Figure 1) [4]. Amyloid plaque is an aggregate of A*β* containing 40–42/43 residues. NFT is primarily composed of hyperphosphorylated form of Tau protein [5]. Tau is synthesized within the neuron and localized in the axon where it promotes stability and assembly of microtubules [6]. During AD progression, tau is hyperphosphorylated and subsequently dissociated from microtubule and polymerized into paired helical filaments (Figure 1) [5, 6]. Although the clinical symptoms of AD are frequently diagnosed in older age, the degenerative process probably starts many years before the clinical onset of the disease [7, 8]. Currently, the diagnosis and treatment of AD is limited. The presymptomatic detection of AD is crucial, as it would facilitate the development of an efficient and rapid treatment of this destructive disorder early on (for recent review see [9–11]).

The biomarkers are the entities whose concentration, presence, and activity are associated with disease. Biomarkers are essential part of disease treatments as they are essential for diagnosis, monitoring the disease progression, detecting early onset of the disease, monitoring the effect of therapeutic intervention, and also avoiding false diagnosis of the disease [19]. An ideal biomarker (1) should be highly specific, (2) should predict the course of illness accurately, and (3) should reflect the degree of response to treatment. The biomarker research for AD has significantly advanced in recent years (Table 1) [9, 10]. The neuroimaging techniques assess the regional structure and function of the brain, as well as assist identifying the biochemical profile of brain dysfunction. The body fluids such as cerebrospinal fluid (CSF), plasma, and urine are considered as important sources for the AD biomarker development (Table 1). CSF is considered a better source for biomarker development as it is in direct contact with the extracellular space of the brain and can reflect biochemical changes that occur inside the brain. Thus far, three CSF biomarkers, A*β*42, total-tau (t-tau), and phosphorylated-tau (p-tau), have been found to have the highest diagnostic potential. Biomarkers of inflammation and oxidative stress and urine-based biomarkers are among the other sources that provide vital information on development and progression of AD. Unfortunately, none of the biomarkers presently available are able to accomplish the disease diagnosis single-handedly. Monitoring more than one biomarker at the same time is suggested to be suitable for detecting the disease progression. The main focus of this paper is to provide insights on the various potential biomarkers with particular emphasis on CSF biomarkers for AD diagnosis. These biomarkers are very promising for early diagnosis of AD.

## 2. Imaging Biomarkers

Neuroimaging techniques provide structural and functional details of the brain immediately [20, 21]. The imaging techniques are also helpful to predict and monitor the disease progression. Recent progress of functional and molecular neuroimaging [22] could provide insights into brain structure and physiology and also could detect the specific proteins and protein aggregates due to AD in the brain [20].

The loss of brain volume is one of the consequences of AD neurodegeneration [11, 21], and it could be differentiated from normal brain by using computerized tomography (CT) and magnetic resonance imaging (MRI) techniques [23]. These techniques are able to show neuronal loss, atrophy of medial temporal regions, as well as neurofibrillary tangles in the brain of AD patients. Using MRI technique, it is now possible to distinguish atrophy during early stage of AD from the atrophy of normal aging [24]. MRI has also the ability to distinguish AD subjects from normal controls, with a very high sensitivity and specificity [24]. MRI can reveal disease progression from cognitive normalcy to mild cognitive impairment (MCI) and to AD [12]. Discrimination of AD from other forms of dementia, namely, frontotemporal dementia (FTD) and DLB (dementia with Lewy bodies) is also possible based on different atrophy patterns that MRI reveals [25–27]. AD is also associated with metabolic impairment with typical regional pattern in the brain and could be detected by positron emission tomography (PET). If 18F-2-deoxy-2-fluoro-D-glucose (FDG) is chosen for PET, the concentrations of tracer imaged then gives tissue metabolic activity, in terms of regional glucose uptake. PET-FDG has been employed to examine regional cerebral metabolism, which is helpful in distinguishing AD from normal patients [13, 28–30]. Recently, several other radiologically contrast compounds have been developed for PET imaging, which could bind the pathological structures such as amyloid plaques, NFT, activated microglia, and reactive astrocytes, enabling the examination of antemortem pathological changes due to AD. The compounds that have been reported as probes for amyloid plaques in PET imaging include, [18F] FDDNP (2-(1-{6-[(2-[F-18] fluoroethyl) (methyl) amino]-2-naphthyl} ethylidene) malononitrile), 18F-BAY94-9172, 11C-SB-13, 11C-BF-227, and 11C-PIB. The only compound developed that can bind NFT *in vivo* is [18F] FDDNP. The 11C-PIB (PIB, Pittsburgh compound B) has been the most extensively studied and applied in AD research [14, 31]. In individuals with AD, increased retention of PIB shows a very specific pattern that is restricted to brain regions (frontal, parietal, temporal, occipital cortices, and striatum), typically associated with amyloid deposition [32]. A significant number of cognitively normal individuals over the age of 60 show a PIB signal pattern indistinguishable from that of individuals with AD, suggesting that measurement of PIB using PET can detect a preclinical stage of the disease. When PIB-PET was performed along with the A*β*42 concentrations in CSF of AD patients, the PIB-positive group showed low A*β*42 levels in the cerebrospinal fluid [33–35]. This finding is consistent with the “amyloid sink” hypothesis [36, 37], according to which the soluble A*β*42 is retained in the brain once plaques are formed. Besides the radiological studies of amyloid plaques and NFT, the PET imaging agent which images the inflammation due to activated microglia and reactive astrocytes has been developed. For example, increased expression of peripheral benzodiazepine receptor (PBR) has been target for the compound [11C] (R)-PK11195. The study using this compound in conjunction with PIB has suggested that microgliosis occurs concomitantly with amyloid deposition and may have direct role in cognitive dysfunction [38]. Like microglia, the changes in astrocytes on association with plaques could be used as biomarkers. For example, using inhibitors of monoamine oxidase B as radiotracers in AD has targeted the elevation of monoamine oxidase B activity [39, 40]. Although all of these imaging techniques are helpful for diagnosis of AD, the approach faced overlapping symptoms due to other pathological processes and normal aging. The approach also needs expensive instruments and experienced personnel for its application in routine diagnosis of AD.

**Fluid Biomarkers**

The sampling of CSF and plasma represents the most direct and convenient means to study the biochemical changes occurring in the central nervous system [10, 19, 41]. These fluids are the most attractive resources for ongoing research for discovering AD biomarkers. Most of the research has been performed either with the plasma or CSF, yet CSF represents more suitable source for biomarker discovery.

## 3. Plasma Biomarkers

Plasma is the liquid portion of blood where red blood cells, white blood cells, and platelets are suspended. Plasma could be easily isolated from whole blood by low speed centrifugation in the presence of an anticoagulant. The easier sampling of blood plasma makes this fluid ideal for biomarker investigation. However, plasma biomarkers as reliable markers for AD have met little success (Table 1). Various blood biomarkers have been proposed, yet changes in the levels of these molecules have proved difficult to verify in independent studies. Multiple studies have identified plasma proteins whose expression levels in AD patients differ from controls. For example, *α*_2_-Macroglobulin (*α*2M) and complement factor H (CFH) showed an increased expression in AD subjects than in control [15]. Both of these proteins are shown to be present in senile plaques [42, 43]. Similarly, the increased levels of *α*_1_-antitrypsin [44], *α*1-antichymotrypsin [45, 46], and decreased levels of Apolipoprotein A1 [47] in blood plasma/serum were observed in AD patients compared to healthy controls. Although these proteins may reflect pathological processes observed in AD and could differentiate diseased plasma compared to controls, these differences have yet to achieve sensitivity, specificity, and reproducibility. Irreproducibility might occur due to different analytical methodologies utilized in various laboratories, different choice of anticoagulant and depletion strategy, and storage related problems. The most popular plasma peptide utilized for biomarker research is A*β*, which is the fundamental element of senile plaques in brain of AD patients [3]. Using ELISA, A*β* can be detected in plasma. The findings from different studies have shown variable results. Some studies have suggested slightly higher A*β*42 or A*β*40 plasma levels in patients with AD than in controls [48]. However, most of the studies have found no change in plasma A*β* concentration between AD and healthy control [48]. It is also suggested that large A*β*42/A*β*40 ratio could indicate the risk factors for AD [49]. These ambiguous results are probably explained by the fact that plasma A*β* is derived from peripheral tissues and does not reflect brain A*β* production. Furthermore, the hydrophobic nature of A*β* makes the peptide bind to plasma proteins, which could result in “epitope masking” [16] and other analytical interferences. Recently, analysis of 18 plasma signaling and inflammatory proteins has accurately identified patients with AD and predicted the onset of AD in individuals with MCI [50]. However, further studies are required to analyze if this set of proteins is the best possible recipe of plasma biomarkers for preclinical AD diagnosis.

## 4. Urine-Based Biomarkers

Neural thread protein (NTP) levels have been consistently identified as an AD biomarker in urine [51, 52]. With disease severity, the urinary concentration of this protein increases. AD associated NTP (AD7c-NTP) in CSF also showed consistent results [51, 53]. More research needs to be done to study the effects of AD7c-NTP levels upon therapeutic intervention [54–56]. Urinary F2-isoprostanes have been reported to be increased [54–56] or unchanged [57, 58], making them less reliable biomarkers. The utility of urine sample for AD diagnosis has advantage that sample collection is relatively easier and noninvasive compared to CSF and plasma. However, very low protein concentrations and high salt levels make it difficult to use urine sample as a source of biomarker [59].

## 5. CSF Biomarkers

Cerebrospinal fluid (CSF) is a translucent bodily fluid that occupies the subarachnoid space and the ventricular system around the brain. CSF acts as a “liquid cushion” providing a basic mechanical and immunological protection to the brain inside the skull and it can be obtained via lumbar puncture. Although lumbar puncture is invasive and potentially painful for the patient, CSF is probably the most informative fluid in biomarkers discovery for neurodegenerative disease prognosis [10]. CSF has more physical contact with brain than any other fluids, as it is not separated from the brain by tightly regulated blood brain barrier (BBB). As a result, proteins or peptides that may be directly reflective of brain specific activities as well as disease pathology would most likely diffuse into CSF than into any other bodily fluid. These proteins and metabolites can serve as excellent biomarkers of AD as well as other neurodegenerative diseases. In early course of AD, for an example of MCI, when the correct diagnosis is most difficult, CSF biomarkers would be valuable in particular [10]. Tau and A*β* in CSF represents the earliest and most intensively studied biomarkers [9, 10, 41, 60, 61]. Both proteins are linked to hallmark lesions of AD, amyloid plaques, and neurofibrillary tangles. In the next section, we will discuss the clinical significance of A*β* and tau biomarkers in detail.

### 5.1. APP, A*β*40/42, and Truncated A*β* in CSF as Biomarkers

One of the major pathological features of AD is the presence of senile plaques primarily composed of A*β*, a proteolytic fragment of the amyloid precursor protein (APP) (Figure 1) [62]. The expression level of APP could serve as diagnostic markers in AD [61]. However, the experimental studies of APP expression level in CSF of AD patients are inconsistent [61]. The inconsistencies between studies ruled out the possibility of CSF-APP being a useful biomarker for AD. APP is expressed in all tissues and could undergo cleavage by either *α*-secretase or *β*-secretase to release sAPP-*α* or sAPP-*β*, respectively. The processing of APP by *α*-secretase occurs via nonamyloidogenic pathway, and a reduced CSF level of sAPP*α* in AD patients has been reported [63]. In contrast, APP processing first by *β*-secretase and subsequent digestion by *γ*-secretase leads to formation of A*β* (38–43 residues) peptides. The 42-residue-long A*β* isoform (A*β*42) is highly hydrophobic and forms oligomers and fibrils that accumulate as extracellular plaques (Figure 1) [4]. Because A*β*42 is the dominant component of the plaques seen in AD [64], many groups have investigated the use of A*β*42, as well as the other A*β* species as a diagnostic tool. The amount of total A*β* in CSF is not well correlated with the diagnosis of AD [65]. The majority of studies have demonstrated a decrease of CSF A*β*42 in AD patients [34, 66–70]. However, few reports suggest the increased [71] or unchanged [72] CSF A*β*42 in AD. These differences in observations might be due to the variations in sample assaying protocols and selection of patient groups. Deposition of the peptide in plaques (“amyloid sinks”) is considered the underlying basis for the decrease of CSF-A*β*42 levels seen in AD [36, 37]. Although it is not clearly proved, the observation is supported by the strong correlation between low CSF-A*β*42 levels and high plaque burden when measured by PIB imaging [33]. This observation was further supported by the fact that AD mouse model showed low CSF A*β* level with high amount of plaque in the brain [73]. Although it has been shown that CSF A*β*42 levels can identify PIB-positive individuals with highest possible sensitivity and specificity, the decreased CSF levels of A*β*42 have also been reported in other dementia such as FTD [74–76]. Low concentrations of CSF A*β*42 was also found with individuals without PIB-positive plaque [77]. This finding might be explained by the fact that PIB binds fibrillar A*β* not the A*β* oligomers or diffuse plaques [77] that are found in earlier stages of AD process. It is however that CSF A*β*42 has high potential as a biomarker for diagnosis, plaque burden, prognosis and may provide clue of preclinical AD. A*β*40 is unchanged in the CSF of AD patients [21]. However, the decreased A*β*42/A*β*40 ratio is much more pronounced in AD diagnosis than the reduction of A*β*42 alone. Therefore, A*β*42/A*β*40 ratio might be more useful in AD diagnosis in the early as well as the clinical phases of the disease [78]. Moreover, the presence of several shorter A*β* isoforms in CSF has suggested that A*β* constitutes a large family of peptides with considerable length variations. The carboxy-terminal truncated A*β* peptides for example, A*β*37, A*β*38, and A*β*39 have been found in CSF of AD subjects. In AD patients, an increase in A*β*38 levels, accompanied with a decrease in A*β*42 levels were also observed [79, 80]. Thus, the A*β*42/A*β*38 ratio might prove useful for more precise diagnosis of AD [79, 80]. Immunoprecipitation techniques and mass spectrometry have identified a number of short truncated A*β* isoforms, such as A*β*14, A*β*15, and A*β*16 in the CSF of AD patients. These forms have been reported to be produced through a novel pathway of APP processing involving the *β* and *α* secretase actions [81]. In the AD subjects, elevated A*β*16 levels, accompanied with a decrease in A*β*42 levels were reported in CSF [82].

### 5.2. CSF-Tau as a Biomarker

The protein tau is an intracellular protein, which maintains the stability of microtubules in neurons. In normal individual, only low concentration of tau is present in CSF. The function of tau is tightly regulated by a number of post-translational modifications including phosphorylation at serine and threonine residues. The precise form of tau in CSF and the mechanism for leakage of intracellular tau into CSF is not clearly understood. Despite intense research, the amyloid and tau pathologies remain unclear. Several experimental studies have suggested that hyperphosphorylation and NFT formation is the downstream phenomenon of AD pathologies [83]. However, it is also noteworthy that loss of function of tau due to hyperphosphorylation and subsequent detachment of tau from microtubule could lead to the increased cytoskeleton flexibility and loss of axonal integrity in the brain (Figure 1) [84]. In AD, tau becomes hyperphosphorylated and gets dissociated from microtubule and subsequently polymerized into insoluble paired helical filaments (PHF) [84]. PHF eventually contributes to the formation of neurofibrillary tangles [85, 86]. NFT formation and neuronal degradation is an essential part of AD pathology (Figure 1). Upon significant disruption of neuronal architecture, tau protein could be released into CSF [60]. Therefore, increased levels of tau and hyperphosphorylated tau in CSF can correlate with the onset of neurodegeneration in AD. The total tau (t-tau) concentration in CSF has been investigated by ELISA analysis using monoclonal antibodies against all tau isoforms. Several studies have suggested that t-tau concentration in CSF of AD patients is higher than control [60, 87]. Although the CSF t-tau is very sensitive biomarker for detecting AD, it has limited ability to discriminate AD from other major forms of dementia as t-tau also increased in CSF of others form of dementia including vascular dementia (VAD) and frontotemporal dementia (FTD) [60]. Several studies also used the p-tau in CSF as potential biomarkers since it is the major component of NFT. CSF concentrations of p-tau in AD have been examined using ELISAs based on monoclonal antibodies that can detect its various epitopes of p-tau, namely, (Thr181 + Thr231), (Thr231 + Ser235), Ser199, Thr231, (Ser396 + Ser404), and Thr181 [41, 88, 89]. ELISA study using all antibodies has showed increased CSF concentration of p-tau in AD patients. Moreover, the ability of increased p-tau assays to discriminate AD from normal aging and other dementia is more sensitive and specific than that of CSF concentrations of t-tau and A*β*42 [60, 90, 91]. The experimental evidences of high CSF concentrations of p-tau in only AD patients have suggested that p-tau is not a simple marker of axonal damage and neuronal degeneration, as t-tau, but it is more closely related to AD pathology and the formation of NFT.

### 5.3. Combined A*β* and Tau in CSF as Biomarkers

It has been suggested that combinations of CSF markers could more successfully discriminate AD from control or other forms of dementia than an individual marker. There are several studies where the diagnostic performance of the combination of CSF t-tau and A*β*42 is analyzed. The evidences have suggested that high CSF concentration of t-tau and low concentrations of A*β*42 could detect AD with high diagnostic sensitivity and specificity [61]. The other combinations of CSF biomarkers have also been evaluated, which suggested that the high CSF p-tau/A*β*42 ratio possesses higher sensitivity and specificity [18] for differentiating AD from normal controls and from subjects with other non-AD dementia than that of the CSF t-tau, p-tau, A*β*42, and ratio of t-tau/A*β*42. It is also suggested that the combination of tau and A*β*42 has more diagnostic potential in terms of sensitivity and specificity in MCI patients to develop future AD [92].

### 5.4. Oligomers of A*β* in CSF: Promising Biomarkers for Early Diagnosis in AD

Recent studies have suggested that oligomeric A*β*s are the most neurotoxic species in AD. Substantial *in vivo* and *in vitro* evidence supports this hypothesis [93–97]. Several *in vitro* neurotoxicity studies have shown that A*β* oligomers are potent neurotoxins [98–104], and the toxicity of some oligomers is higher than that of the corresponding amyloid fibrils [105]. The evidences, which support the fact that A*β* oligomers could be targeted for drug and biomarker discovery include (1) soluble oligomers could inhibit hippocampal long-term potentiation (LTP) [95, 98, 100, 103, 104, 106, 107] and disrupt cognitive function [108] *in vivo*; (2) compounds that bind and disrupt the formation of oligomers have been shown to block the neurotoxicity of A*β* [108, 109]; (3) drugs that reduce the amyloid plaque burden without disruption of oligomers have little effect on recovery of neurological function [110]. Many oligomers such as A*β*-derived diffusible ligand (ADDL)-like A*β*42 oligomers [111], 90 kDa A*β*42 oligomer [112, 113], 56 kDa oligomer of “Aβ*56 [114], and A*β* trimers [115] have shown high *in vivo* toxicity, providing a compelling reason for A*β* oligomers to be used as potential AD biomarkers especially for early diagnosis in AD. In addition, elevated levels of A*β* oligomers were detected in AD patients and transgenic mice compared to control [116–118]. The elevated level of oligomers could also appear in CSF but with lower concentration. Therefore, highly sensitive techniques are required for oligomer detection in CSF. The study using fluorescence correlation spectroscopy suggested the presence of A*β* oligomers in CSF of AD patients, compared to healthy control [119]. Recently, ultrasensitive, nanoparticle-based, protein detection assay (bio-barcode) showed that the ADDLs concentrations in CSF of AD patients were consistently higher than the nondemented age-matched control [120]. In this study, ADDLs specific antibodies coupled to DNA-tagged nanoparticles were used to capture the oligomers from the CSF of patients with AD. Although the number of AD patients and controls studied was low, the findings were very promising. Although A*β* oligomers are attractive biomarker candidates, several limitations exist to use these species. The concentration of these A*β* oligomers in CSF is very low in comparison with A*β* monomers. Again, the detection of individual A*β* oligomers is difficult since oligomers are metastable and therefore one form of oligomers could transform to another form immediately. Assay sensitivity must reach very high level if one can detect total heterogeneous population of A*β* oligomers in CSF. The monoclonal antibodies specific for only A*β* oligomers could be difficult to develop. Recently, an antibody against A*β* oligomers was developed, which can detect all A*β* oligomers, including oligomers from other amyloidogenic protein [117]. However, using these oligomers specific antibody to diagnose of AD is difficult since it cannot differentiate AD from other neurodegenerative diseases. New analytical methods and novel oligomers-specific antibody must be developed to detect oligomers in CSF of AD patients, which would have ultimate ability to detect early onset of AD.

### 5.5. Neuronal Biomarkers in CSF

Besides tau and A*β*, neuronal and synaptic proteins could also be used as CSF biomarkers in AD. For example, Visinin-like protein 1 (VLP-1), a calcium sensor protein was shown to be significantly increased in the CSF of AD subjects compared to controls. It is believed to seep out from dented neurons [121]. The sensitivity and specificity of CSF VLP-1 is comparable to CSF t-tau, p-tau, and A*β*42. Combined analysis of A*β*42, p-tau, and VLP-1 has been reported to raise the diagnostic precision of AD. VLP-1 biomarker might also prove useful in indicating the degree of dementia [121]. The neurofilaments, which are structural component of axons, could also be used as biomarkers for discriminating AD patients from other forms of dementia, as their expression levels are high in VAD and FTD [122], while normal levels are found in most AD patients. Another synaptic protein called growth-associated protein (GAP-43) is found in higher levels in CSF of AD than that of controls, and FTD [123]. Furthermore, it has been shown that CSF GAP-43 and t-tau were increased in AD and correlated positively [123], suggesting both biomarkers are reflecting axonal and synaptic degeneration.

### 5.6. Oxidative Stress Marker in CSF

Besides the formation of amyloid plaque and NFT, AD is also frequently characterized by reactive oxygen species (ROS)-mediated neuronal damage. The oxidative damage in the brain mainly involves lipid peroxidation [124]. Polyunsaturated fatty acids are susceptible to oxidation by reactive oxygen species. Isoprostanes are lipid oxidation products generated due to the reaction between fatty acids and ROS. Therefore, isoprostanes could be used as valuable AD biomarkers. Several studies have suggested that F2-isoprostanes, a group of isoprostanes, are increased in CSF of AD patients compared to healthy control or patients with other dementia [125, 126]. CSF-F2-isoprostanes have also been shown to be increased in patients with MCI and asymptomatic carriers of familial AD mutations. A combined analysis of CSF-A*β*42, tau, and F2-isoprostanes, was able to diagnose AD with a sensitivity of 84% and specificity of 89% [127].

### 5.7. Inflammatory Biomarkers in CSF

AD pathology involves release of inflammatory mediators. The differential occurrence of several proteins due to inflammatory process in AD might be used as biomarker. These proteins can be detected using ELISA, as well as proteomics approaches. One of the most studied inflammatory biomarkers is *α*1-antichymotrypsin (A1ACT), which is observed either increased [45, 128] or unchanged [129] in CSF samples of AD patients. However, the contradictory results suggest that more studies must be conducted to raise the possibility of A1ACT to be regarded as an effective biomarker. The study of cytokines, which are produced during inflammation processes in AD, also gave inconsistent results. For example, CSF interleukin-6 (IL-6) levels have been reported to be increased [130–132], decreased [133], or unchanged [134–136] in AD. Studies of IL-6 receptor, Gp130, and tumor necrosis factor (TNF-*α*) also produced conflicting results [137]. The genetic background, environmental factors, and usage of anti-inflammatory drugs might produce substantial variation in cytokine levels in an individual [138]. This could be the reason for such uncertain results.

## 6. CSF Biomarkers: A Potential Hope for AD Diagnosis

As discussed in the preceding sections, most biomarker research in AD is based on either brain imaging or is fluid-based. Although imaging techniques are definitive tests for detecting amyloid plaques and atrophy using molecular probe, still antemortem diagnosis of AD and MCI are less successful. More sensitive chemical probes are required to be developed, which would bind oligomers or diffuse plaque. However, imaging techniques being very expensive and requiring more experience for handling the instruments precludes their day-to-day use for AD diagnosis. In fluid biomarker research, CSF has been proved to be a supreme source for biomarkers for several reasons. CSF is in close proximity to the brain, and therefore biochemical changes in the brain affect the composition of biomarkers in CSF. Since AD pathology is restricted to the brain, CSF is an obvious source of biomarkers for AD. CSF is also a rich source of brain-specific proteins, and changes in these protein levels are observed in CSF with disease progression. CSF biomarkers are also very sensitive to the fine changes in brain that occur in the preclinical stages of the AD. Therefore, CSF is probably the most informative fluid sample available for preclinical as well as symptomatic AD diagnosis. The diagnostic sensitivity and specificity of CSF biomarkers in differentiating AD from healthy controls, and from other forms of dementia is already achieved with satisfactory levels. Moreover, a combination of more than one biomarker in CSF, such as CSF p-tau, t-tau, and A*β*42 is considered to give higher diagnostic accuracy of AD. It can identify AD, prodromal AD, and also can differentiate AD from other dementia with high sensitivity and specificity that is otherwise impossible to achieve.

Although CSF biomarkers have proved to be highly informative, sensitive, and specific for detection of clinical AD and early stage of AD, their regular use in clinic is still limited. One of the major reasons against the vast applicability of CSF in AD diagnosis is lumbar puncture, an invasive method to collect the CSF sample. Other issues including inconsistency of data analysis of CSF sample due to sample collection, transportation, storage, and high expense of the test might limit the use of CSF for routine diagnosis. However, various strategies are available to resolve these issues. For example, the Clinical Neurochemistry Laboratory in Gothenburg, Sweden and Alzheimer's Association, have together started a quality control program, the objective of which is to standardize CSF biomarker measurements between both research and clinical laboratories [10]. This program would obviously enhance the diagnostic precision of CSF markers, thus enabling them to support a routine analysis for diagnosis of AD.

## 7. Future Direction

According to the current clinical diagnostic criteria, AD diagnosis cannot be made until the patient has dementia, which is defined as cognitive symptoms severe enough to interfere with social or occupational activities [139]. This might hinder the preclinical diagnosis of AD. The disease modifying drugs will be most effective and will have most therapeutic value if these are administered in the earliest stage of AD, before amyloid plaques and NFT become prevalent. Since AD is a multifactorial neurodegenerative disorder both at clinical and neuropathological level, development of biomarkers with 100% efficiency in terms of sensitivity and specificity is difficult to achieve. Also, the effectiveness of the disease modifying drugs could vary from one subgroup to another subgroups, making the utility of biomarkers in clinical trial and drug discovery difficult. Combined analysis of CSF biomarkers represents more suitable diagnostic tool to detect AD patients or detect individuals with MCI. Moreover, sensitive assays should be developed to detect amyloid oligomers in CSF and in the brain. This would raise the possibility for the diagnosis of early onset of AD.

## Figures and Tables

**Table 1:** Some promising biomarkers in diagnosis of AD.

Category	Markers	Advantages	Limitations	References
Imaging		(1) Noninvasive	(1) Expensive	[12–14]
	CT, PET, PIB-PET,	(2) Provides structural and functional	(2) Requires experienced personnel	
	MRI	details of brain immediately	(3) The sensitivity and specificity to	
		(3) Can reveal disease progression	AD is not satisfactory	

Plasma	*α*_2_-Macroglobulin,	(1) Noninvasive	(1) Less correlation to AD	[15–17]
	Complement	(2) Samples are easily accessible	(2) Less sensitive and specific for AD	
	factor H, A*β*42		diagnosis (due to epitope masking)	

CSF	A*β*42, t-tau,	(1) Can correlate AD directly	(1) Invasive, sample has to be collected	[10, 18]
	p-tau p-tau/A*β*42,	(2) Highly sensitive and specific	by lumbar puncture	
	t-tau/A*β*42	(3) Can detect AD progression	(2) Irreproducible diagnosis due to	
			sample storage and transportation	

## Abbreviations

A*β*: Amyloid *β* protein
AD: Alzheimer's disease
ADDL: A*β*-Derived diffusible ligand
APP: Amyloid precursor protein
BACE: *β*-site APP-cleaving enzyme
BBB: Blood brain barrier
CT: Computerized tomography
DLB: Dementia with Lewy bodies
FTD: Frontotemporal dementia
MCI: Mild cognitive impairment
MRI: Magnetic resonance imaging
NFT: Neurofibrillary tangles
PET: Positron emission tomography
PIB: Pittsburgh compound B
p-tau: Phosphorylated-tau
VAD: Vascular dementia.
